# Type 1 Autoimmune Pancreatitis in a Teenager With Ulcerative Colitis: A Case Report

**DOI:** 10.1016/j.gastha.2025.100834

**Published:** 2025-10-10

**Authors:** Jerry H. Rose, Michael J. Sikorski, Patrick W. Ruane, Dania Hudhud, Abdulhameed M. Al-Sabban

**Affiliations:** 1 Division of Gastroenterology and Hepatology, Department of Medicine, University of Maryland School of Medicine, Baltimore, Maryland; 2 Department of Pediatrics, University of Maryland School of Medicine, Baltimore, Maryland

**Keywords:** Autoimmune Pancreatitis, Pediatrics, Type 1 AIP, Subclass, Pancreatic Disease

## Abstract

Type 2 autoimmune pancreatitis (AIP) is a recognized complication of ulcerative colitis (UC), whereas type 1 AIP—an immunoglobulin G4-related sclerosing pancreatitis—is uncommon in this patient population and typically presents in older males. We present a case of recurrent pancreatitis in an 18-year-old male with UC. Investigations revealed elevated immunoglobulin G4 levels and images demonstrating diffuse pancreatic strictures and parenchymal enlargement, consistent with type 1 AIP. The patient showed a favorable response to glucocorticoid therapy. This case highlights a potential new association between AIP and UC and emphasizes early recognition and treatment.

## Introduction

Autoimmune pancreatitis (AIP) is a rare form of acute pancreatitis that can progress to chronic pancreatitis. Histological profiling reveals two distinct disease populations: type 1, lymphoplasmacytic sclerosing pancreatitis, and type 2, idiopathic duct-centric pancreatitis. Type 1 AIP is associated with immunoglobulin G4 (IgG4)-related diseases and classically presents in older males (mean age, 61.4 years) with obstructive jaundice (63% in type 1 vs 8.4% in type 2),^1^ elevated serum IgG4 levels, and potential multi-organ involvement. Type 2 AIP typically presents in younger patients (33.5 years)^2^ with signs of acute pancreatitis (34%–64% in type 2 vs 5% in type 1),^1^ normal serum IgG4 levels, and is characterized by neutrophilic injury to the pancreatic ducts. It is associated with ulcerative colitis (UC) in approximately 48% of cases.^3^ Type 1 AIP is rarely seen in UC.^1^ To our knowledge, this is the third case report implicating type 1 AIP in young patients with UC.^4^^,^^5^ Each patient was a teenager who was first diagnosed with UC and later developed recurrent pancreatitis. One patient did not respond to steroids due to necrotizing pancreatitis, so a distal pancreatosplenectomy was conducted to control symptoms.^4^

## Case Report

An 18-year-old male with a history of biopsy-confirmed UC proctitis diagnosed 6 months prior, treated with oral and rectal mesalamine, and in endoscopic remission, presented with severe epigastric pain for 3 days. His symptoms began 1 month prior and were described as intermittent, “stabbing or throbbing,” non-radiating, and worse with eating, leading to fatigue and a 10-pound weight loss.

At an outside hospital, the patient was diagnosed with acute pancreatitis, with a markedly elevated lipase level (>4000 U/L, reference range: 23–300 U/L). Mesalamine was initially held as a possible cause but was resumed after markedly elevated IgG4 levels (2430 mg/dL, reference range of: 4.0–86.0 mg/dL) and magnetic resonance cholangiopancreatography (MRCP) findings supported a diagnosis of presumed type 1 AIP. Prednisolone 10 mg daily was initiated.

**Figure 1:**
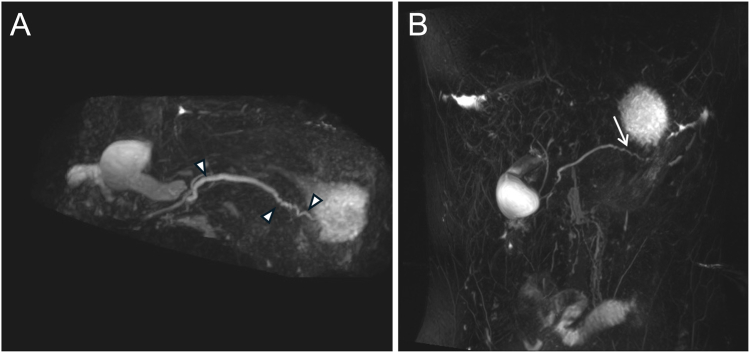
Magnetic resonance cholangiopancreatography images obtained (A) before and (B) after steroid therapy reveal a reversal of pancreatic duct narrowing. (A) Arrowheads point to strictures throughout the pancreatic duct. (B) Arrow points to normal pancreatic duct appearance at the tail.

**Figure 2:**
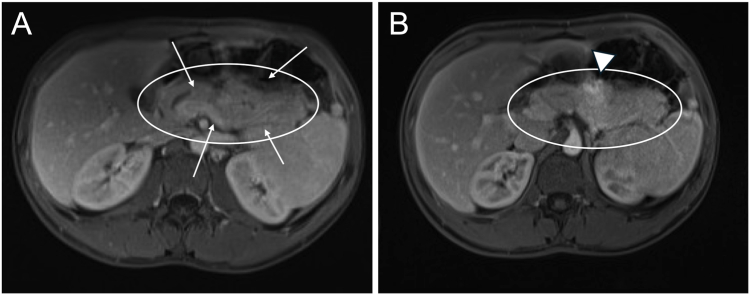
Magnetic resonance imaging of the abdomen obtained (A) before and (B) after steroid therapy reveals interval atrophy of the gland with resolution of the peripancreatic hypodense rim. Circles outline the pancreas. Arrows point to diffuse parenchymal swelling (sausage-shaped pancreas), which persists. Arrowhead points to a focal inflammatory mass.

On our examination, he appeared underweight with a body mass index of 17.7 kg/m^2^ (2nd percentile). Abdominal palpation elicited epigastric and generalized abdominal tenderness. Laboratory evaluation revealed elevated serum lipase (5573 U/L) and serum IgG4 (153.2 mg/dL). MRCP revealed features suggestive of acute pancreatitis involving the distal body and tail. There was an irregular pancreatic duct narrowing at the tail and a dilated main pancreatic duct with an abrupt cutoff at the head (Figure 1A). Global parenchymal enlargement resulted in a “sausage-shaped” pancreas (Figure 2A). No gallstones were identified on cross-sectional imaging. Prior MRCP studies were unavailable for comparison. The key case findings are summarized in Table.

**Table:** Timeline of Clinical Presentation, Diagnostic Findings, Treatment Interventions, and Outcomes

Time from presentation	Location	Findings/Results	Outcome/Next step
0 wk	Initial presentation at outside hospital	Severe epigastric pain ×3 d, fatigue, 10–lb weight loss; lipase >4000 U/L; IgG4 2430 mg/dL; MRCP consistent with type 1 AIP	Diagnosed with acute pancreatitis; mesalamine held; prednisolone 10 mg started
4 wk	Recurrence of symptoms at our hospital	Epigastric and generalized abdominal tenderness; lipase 5573 U/L; IgG 153.2 mg/dL; MRCP: distal body/tail pancreatitis, irregular duct narrowing, dilated main pancreatic duct with cutoff at head; “sausage-shaped” pancreas magnetic resonance imaging	Prednisone 40 mg × 2 wk, then 7-wk taper
7 wk	Post-treatment follow-up	No evidence of acute pancreatitis	Clinical improvement
∼8 wk	Inflammatory bowel disease clinic visit	Flare of ulcerative colitis (abdominal pain, bloody mucoid diarrhea)	Oral mesalamine increased from 2.4 g → 4.8 g daily
Latest follow-up	Ongoing management	No recurrence of pancreatitis; maintained on low-dose prednisone	Stable clinical course

The patient tolerated a diet and was discharged the following day on prednisone 40 mg daily ×2 weeks, followed by a slow taper over seven additional weeks. Three weeks after discharge, a repeat MRCP showed no evidence of acute pancreatitis (Figures 1B and 2B). However, the patient began experiencing a UC flare and was seen in the outpatient inflammatory bowel disease clinic. Oral mesalamine was adjusted to 2.4 g daily and later increased to 4.8 g daily to treat subsequent UC flares. He underwent an otherwise negative rheumatology workup for myeloperoxidase antineutrophil cytoplasmic antibody (MPO-ANCA), proteinase-3 antineutrophil cytoplasmic antibody (PR3-ANCA), antinuclear antibody, immunoglobulin subclass, and complement levels. Serological testing for hepatitis B, hepatitis C, HIV, rapid plasma reagin, and tuberculosis was negative.

He continues maintenance on low-dose prednisone 2.5 mg daily. Pancreatitis has not recurred in 8 months since discharge from the hospital.

## Discussion

### Epidemiology

Multiple criteria have been proposed to define AIP. In 2011, the International Consensus Diagnostic Criteria for AIP was developed to aid clinicians in diagnoses.^6^ AIP can be probable or definitive based on five cardinal features: imaging of pancreatic or ductal changes, serology, other organ involvement, histology, and response to steroid therapy. A combination of features suggests AIP. Definitive type 1 diagnoses can be made in the absence of histology.^6^ Elevated serum IgG4 (>140 mg/dL) has 72% sensitivity and 93% specificity for diagnosing type 1 AIP.^7^ Type 2 requires histology, showing an inflammatory cell infiltrate around ductal epithelium of neutrophils and scant (<10) IgG4+ plasma cells/high power field, to make a definitive diagnosis.^6^

With increased recognition, AIP incidence doubled between 2011 and 2015.^8^ The estimated prevalence is 10.1 per 100,000 population.^1^ Most cases have been reported in East Asia. Serum IgG4 levels are significantly higher in patients with extrapancreatic lesions.^1^ Pancreatic tissues are obtained in 60% of patients. Patients with inflammatory bowel disease develop AIP at a rate 400 times greater than the general population.^8^^,^^9^

### Diagnostic Considerations

Our patient meets International Consensus Diagnostic Criteria for definitive type 1 AIP.^6^ These include imaging features of multiple pancreatic duct strictures without upstream dilation and diffuse parenchymal enlargement, response to steroid therapy, and serum IgG4 levels 1–2 times the upper limit of normal.^6^^,^^10^

While type 2 AIP is more commonly associated with UC, our patient presented with features characteristic of type 1 AIP. This case underscores the importance of considering type 1 AIP in young patients with UC and recurrent pancreatitis. There is an overlap between the AIP subtypes in our report and those in two similar reports.^4^^,^^5^ The diagnosis of pediatric AIP relies on adult criteria. However, the distinction between AIP subgroups may not apply to children.

### Treatment Strategies

Glucocorticoids remain the primary treatment for AIP. Although induction therapy is agreed upon, there is no evidence-based recommendation regarding maintenance therapy.^11^ The optimal dose and duration have not been evaluated in a clinical trial.^12^ Initial therapy consists of prednisone 0.6–1 mg/kg with reassessment of imaging, serum IgG4, and CA19-9 2 weeks after treatment. Low-dose glucocorticoid maintenance monotherapy at 2.5–7.5 mg/day for up to 3 years prevents relapses in type 1 AIP^11^ (31% of patients experience at least one relapse).^1^ Following these guidelines, we induced remission in our patient.

## Conclusion

The case presented highlights a unique association of type 1 AIP that developed in the setting of UC. Narrow definitions of autoimmune conditions may not be appropriate. Given the overlap of AIP subtypes, a broad differential for new pancreatitis in UC patients should include IgG4 levels.
